# Corrosion behavior of an AZ91D magnesium alloy under a heterogeneous electrolyte layer

**DOI:** 10.1371/journal.pone.0234981

**Published:** 2020-06-23

**Authors:** Yanhua Wang, Cheng Zhen, Zhan Zhang, Zhencheng Tao

**Affiliations:** 1 Key Laboratory of Marine Chemistry Theory and Technology, Ministry of Education, College of Chemistry and Chemical Engineering, Ocean University of China, Qingdao, China; 2 Library of Ocean University of China, Qingdao, China; 3 Key Laboratory of Marine Ecology and Environmental Sciences, Institute of Oceanology, Chinese Academy of Sciences, Qingdao, China; 4 Laboratory for Marine Ecology and Environmental Science, Qingdao National Laboratory for Marine Science and Technology, Qingdao, China; 5 Center for Ocean Mega-Science, Chinese Academy of Sciences, Qingdao, China; College of Engineering, University of Saskatchewan, CANADA

## Abstract

The corrosion behavior of an AZ91D magnesium alloy was investigated under a heterogeneous electrolyte layer by using electrochemical methods and surface analysis techniques. Dynamic polarization curves and morphological characterization were obtained at the center and near the edge zones under the electrolyte layer. The influence of the gas/liquid/solid three-phase boundary zone (TPB) on the corrosion behavior of the AZ91D magnesium alloy was discussed. The corrosion rate changed more significantly near the TPB zone than that at the other zones. The AZ91D alloy exhibited the characteristics of filiform corrosion together with shallow pitting corrosion. Different from the randomly distributed shallow pits, the filiform corrosion preferred to initiate near the TPB region and then progressively expanded adjacent to the edge of the electrolyte layer. The TPB zone played a vital role in determining the corrosion location, the corrosion morphologies and the corrosion rate of the magnesium alloy by influencing the mass transport process of carbon dioxide.

## 1 Introduction

A marine atmospheric environment has high humidity and contains a great amount of hygroscopic salts (mainly NaCl) generated from sea spray. Due to rainfall or dew condensation, the hygroscopic salts can deposit and develop thin electrolyte layers (TELs) on metal surfaces. As the lightest engineering metal, magnesium alloys are often used in applications where they are exposed to ambient marine atmospheres and usually suffer serious atmospheric corrosion under TEL [1] [2] [3] [4].

Several papers have reported the corrosion behavior of magnesium alloys under TELs [5] [6] [7] [8] [9] [10] [11]. Most of the studies have investigated the influence of TEL thickness on the corrosion behavior of magnesium and its alloys [8] [9] [10]. They found that localized corrosion is the primary form of corrosion, which is in a higher ratio compared to that of uniform corrosion [11]. These studies have focused on the corrosion behavior of magnesium alloys under a homogeneous electrolyte layer.

Some studies have concentrated on the corrosion behavior of magnesium alloys under an adsorbed film in ambient atmosphere. They studied the corrosion of magnesium alloys in humid air and indicated that the relatively benign behavior of Mg-Al alloys in atmospheric corrosion has been attributed partly to the inhibitive effect of carbon dioxide [5] [6] [7]. Most of the work was investigated by the gravimetric method and surface analysis technique, with an emphasis on the influences of the microstructure.

These thin water films are heterogeneous and exhibit a gas/liquid/solid three-phase boundary (TPB) owing to the existence of surface tension. The TPB zone refers to a narrow liquid zone connecting the three phases and plays an important role in the corrosion process [12]. Compared with the general reaction area of the liquid phase, the TPB zone is a zone with high-speed cathodic reactions in which the diffusion rate of oxygen is much higher than in the bulk solution [13]. For the corrosion of Mg alloys, the main cathodic reaction is the reduction of hydrogen ions, so the reaction mechanism and mass transfer process are different.

To date, studies on the corrosion behavior of Mg alloy have been less reported under a heterogeneous electrolyte layer, and the influences of the TPB zone are especially unclear. The aim of the present research was to investigate the corrosion behavior of magnesium alloys under a heterogeneous electrolytic layer and, on this basis, to clarify the influences of the TPB zone.

## 2 Material and method

### 2.1 Preparation of the electrodes

The material used was as-cast AZ91 magnesium alloy (Shanxi Magnesium Co., Ltd, China), with an exposed area of 10 mm×10 mm in this experiment. The specimens were sealed by epoxy resin after adding an electrical connection with copper wires. Then, the electrodes were mechanically ground with SiC papers up to No. 2000 grit, cleaned ultrasonically with ethanol, and then rinsed with deionized water. Before placing the electrolyte layer on the surface, the electrodes were stored in a desiccator for approximately 24 hours.

The silver/silver chloride (Ag/AgCl) electrode was obtained by an electrochemical chlorination method [14]. First, the silver chloride was precipitated by electrochemically chlorinating the silver wire (Φ 200 μm, purity 99.9%, Sinopharm Chemical Reagent, China) in a 1 mol/L potassium chloride solution (Tianjin Bodi Chemical Reagent, China) under a 1 mA/cm^2^ anodic current for 1 hour. After that, the electrode was cleaned with deionized water and treated at 280°C in a muffle furnace for 2 hours. The Ag/AgCl reference electrode was formed by inserting the chlorinated silver wire into a micropipette (outer tip diameter, 100 μm), which was filled with a gel consisting of 2 wt.% agar (Sinopharm Chemical Reagent, China) dissolved in a saturated potassium chloride solution. The potential of the electrode was calibrated by a saturated calomel electrode (SCE), and all potentials reported in this work were converted to values relative to SCE.

**Fig 1:**
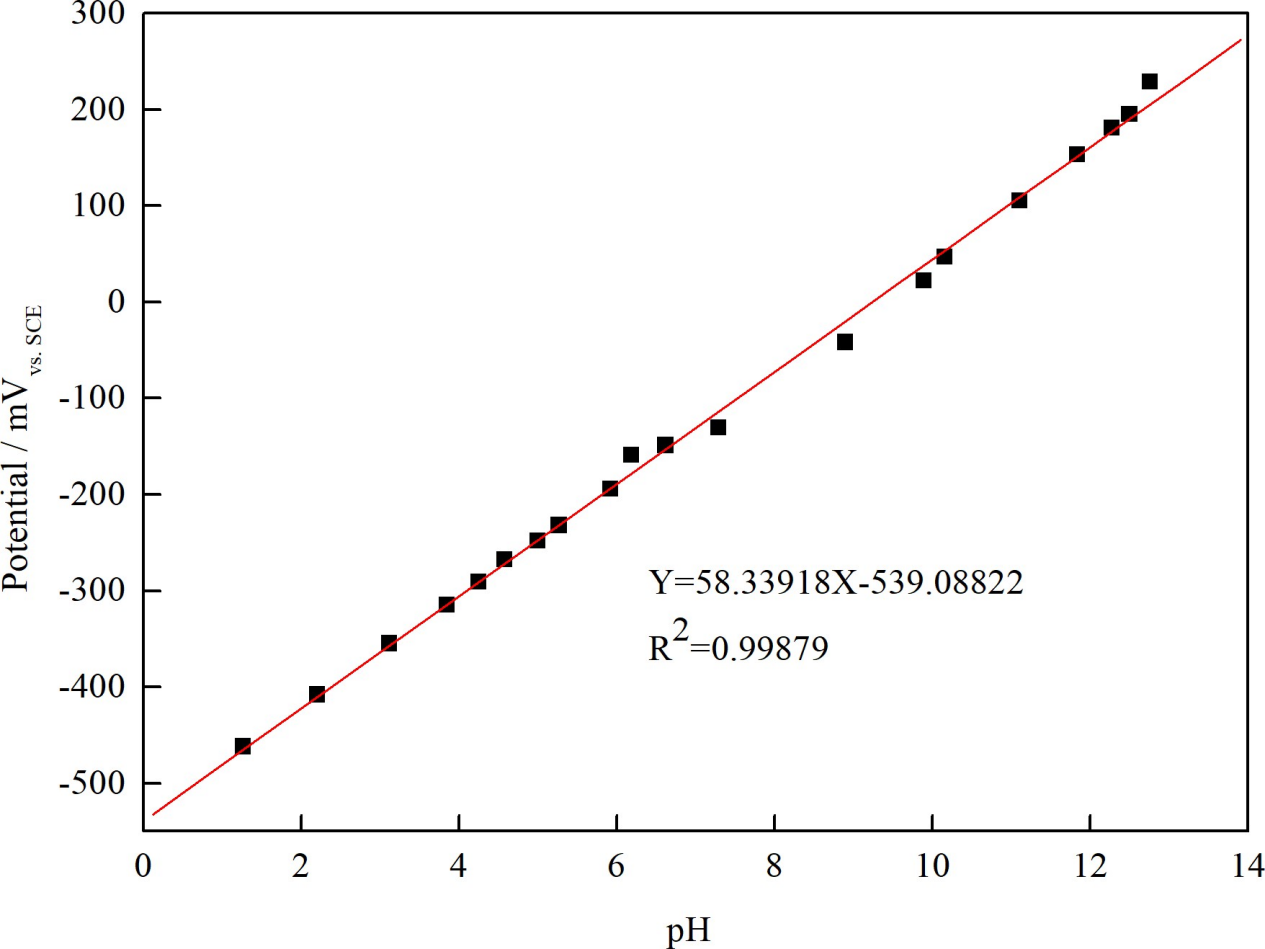
Variations of potential with pH for the Ir/IrO_2_ electrode.

An iridium/iridium oxide (Ir/IrO_x_) electrode was fabricated by oxidation of an Ir wire (Φ 500 μm, purity 99.7%, Shanghai Shenjie Instrument, China) in a sodium carbonate melt at 820°C in a muffle furnace for 5 hours. Then, it was immersed in 1 mol/L hydrochloric acid to remove the residual carbonates and rinsed with deionized water. Next, the wire electrode was treated at 120°C in an oven for 12 hours to obtain the Ir/IrO_x_ electrode [15]. The pH value was obtained by measuring the potential difference relative to the above-mentioned Ag/AgCl reference electrode. As shown in Fig 1, the homemade Ir/IrO_2_ electrode showed a good linear response to pH and the slope was measured to be 58.34 (close to the theoretical value 59.13).

### 2.2 Electrochemical measurements

A thin electrolyte layer (40 μL, 0.6 mol/L NaCl, Sinopharm Chemical Reagent, China) was placed on the surface of the Mg alloy by using a syringe. The exposed surface area to the NaCl solution was about 0.36 cm^2^. Then, the samples were placed in a temperature-humidity test chamber where the humidity was controlled using sodium chloride solution. The experiments were carried out at 20.0±0.5°C, a relative humidity 93±2%, and the atmosphere of the laboratory with a carbon dioxide concentration of approximately 500±30 ppm.

**Fig 2:**
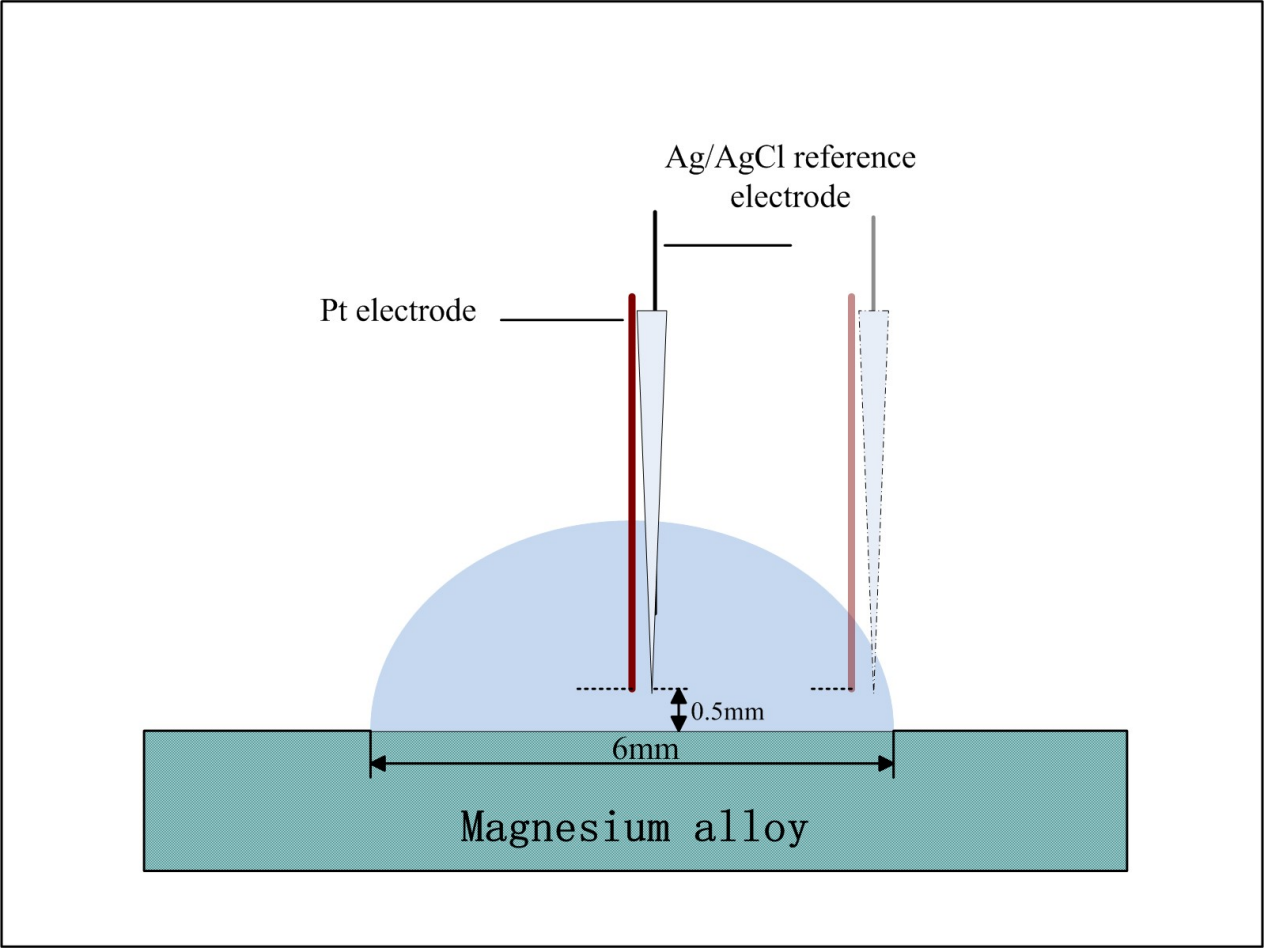
The schematic diagram of the electrochemical measurement.

Potentiodynamic polarization curves were obtained through an electrochemical system PAR S2263 (Princeton Applied Research, USA). For all electrochemical tests, a three-electrode system was employed with the AZ91D alloy as the working electrode, Pt wire (Φ500 μm, Sinopharm Chemical Reagent, China) as the auxiliary electrode and a homemade Ag/AgCl electrode as the reference electrode. The samples were kept in open-circuit potential (OCP) conditions for more than 30 minutes before starting the polarization measurements. The polarization curves were recorded with a rate of 10 mV/s from -1.8 V to -1.0 V at the center and near the TPB zone, respectively. During the measurements, the Ag/AgCl reference electrode and Pt auxiliary electrode were positioned approximately 500 μm above the sample surface underneath the electrolyte layer. The schematic diagram of the electrochemical measurement was shown in Fig 2.

### 2.3 Surface analysis

The specimens were cleaned with deionized water and dried with cold air after the exposure test. Then, the morphologies of the specimens were observed by scanning electron microscopy (SEM, model JSM-6700FX, Japan), and the chemical compositions of the relevant zones were obtained by energy dispersive spectrometry (EDS, Oxford X-MAX, UK). X-ray photoelectron spectroscopy (XPS) analysis was carried out using ESCALAB 250 XI. The X-ray source was the Ka peak of aluminum. Avantage software was used to analyze the data. The accurate binding energies were determined by referencing to the adventitious C1s peak at 284.8 eV.

## 3 Result

**Fig 3:**
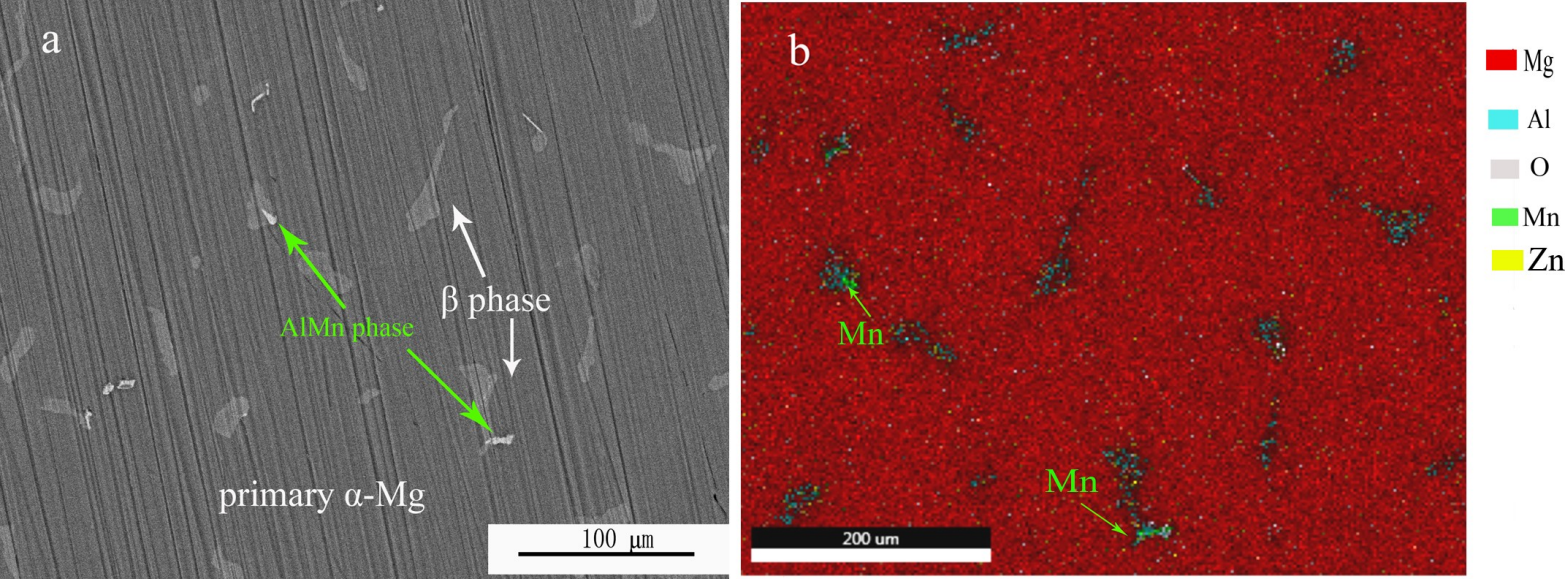
SEM (a) and EDS (b) images of AZ91D magnesium alloy before corrosion exposure.

The microstructure of AZ91D magnesium alloy was measured before corrosion exposure. As shown in Fig 3, AZ91D magnesium alloy exhibited a multiphase microstructure, consisting of primary α-Mg phase, β phase (Mg_17_Al_12_ phase) and AlMn phase precipitates (indicated by arrows). From the element distributions, the main element was Mg and Al together with a small amount of O, Zn and Mn. The element Al was mainly precipitated in the form of β phase accompanied with AlMn particles. While the distribution of element O and Zn was relatively uniform on the surface of magnesium alloy.

### 3.1 Potentiodynamic polarization

**Fig 4:**
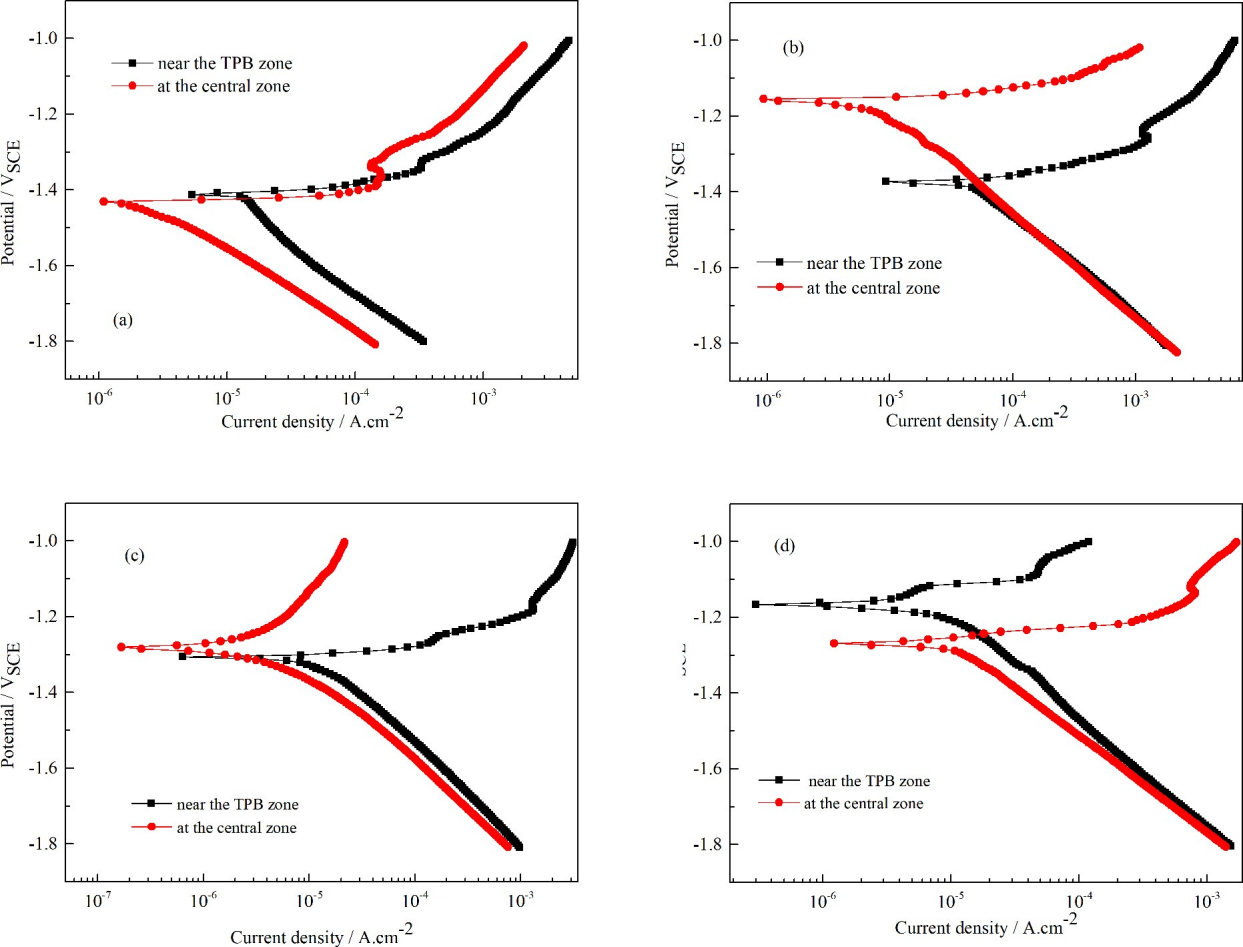
Typical polarization curves of AZ91D alloy under TEL for different times. a: 45 min, b: 3 h, c: 6 h, d: 12 h.

The corrosion behavior of the AZ91D alloy was investigated under the TEL at the center and near the TPB regions, respectively. Fig 4 shows the typical polarization curves of the AZ91D alloy under the TEL at different times. At the initial stage, both the anodic and the cathodic current density near the TPB region were larger than those at the central region (Fig 4A). With increasing corrosion time, the cathodic polarization curves almost overlapped, while the anodic curves were quite different (Fig 4B, 4C and 4D). This implied that the differences in the corrosion behavior between the central and TPB regions were probably due to the anodic reaction process. Compared with the anodic curves at the central region, the TPB region exhibited high anodic current density for the first 6 hours but low current density after exposure for 12 hours.

**Fig 5:**
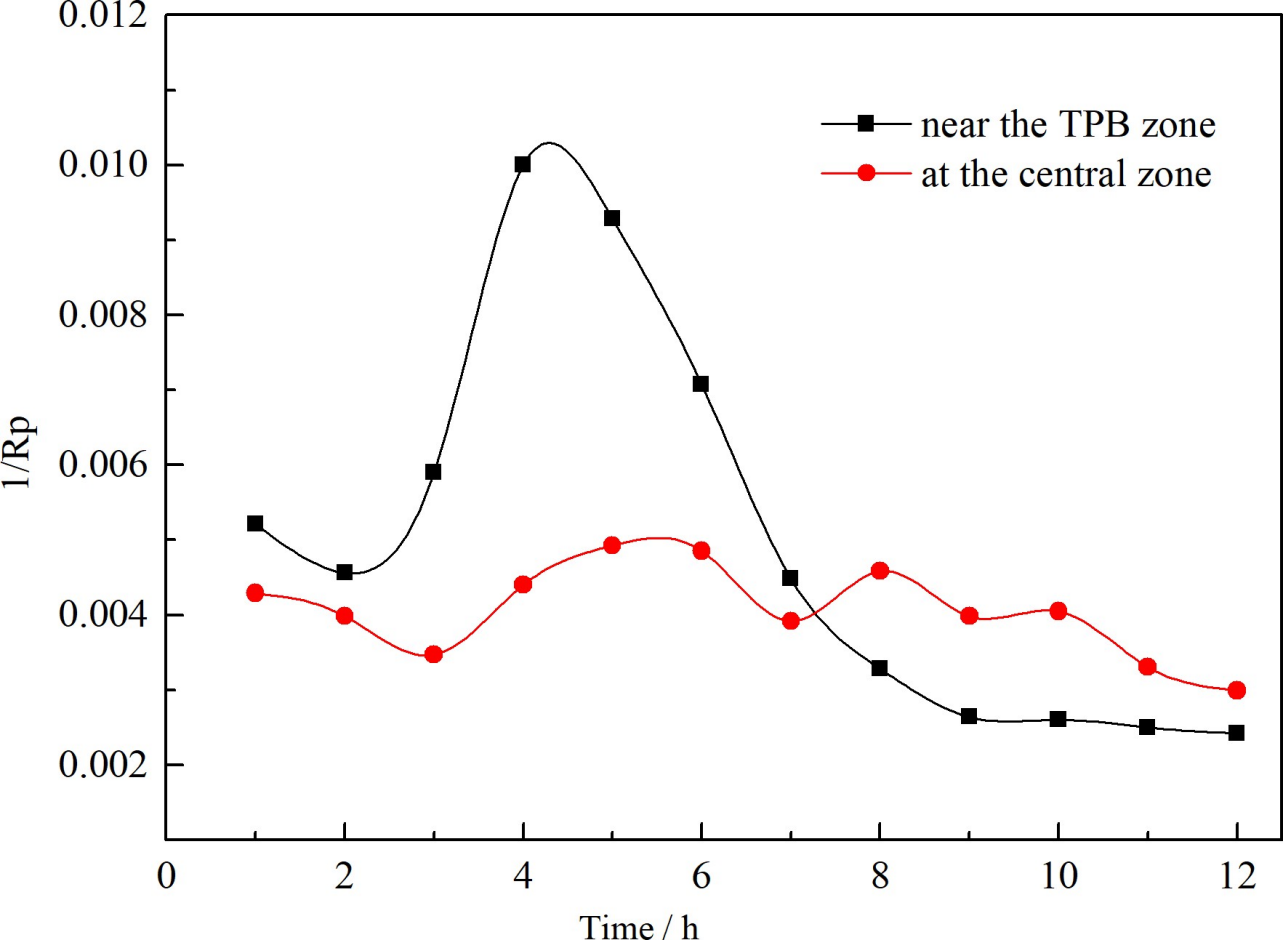
Variations of 1/R_p_ with the exposure time.

To compare the corrosion tendency at different regions under a heterogeneous electrolyte layer, the variation of 1/R_p_ with time was shown in Fig 5. The polarization resistance R_p_ was obtained from the linear polarization measurements, and the value of 1/R_p_ was used to represent corrosion rate according to the Stern-Geary equation (i_corr_ = B/ R_p_). As can be seen, 1/R_p_ initially increased and then decreased near the TPB zone, reaching a maximum value after several hours. While, 1/R_p_ measured at the central region exhibited fluctuations within a narrow range. Comparatively, the corrosion rate was more stable at the central zone than near the TPB zone.

It should be noted that it is impossible to obtain the static polarization curves using a high scan rate and a small sample area. However, instead of obtaining the true corrosion rate, the present work aims to compare the change tendency of different regions on a Mg alloy under the TEL, which helps to understand the influences of the heterogeneous electrolyte layer.

### 3.2 Variations of pH value

**Fig 6:**
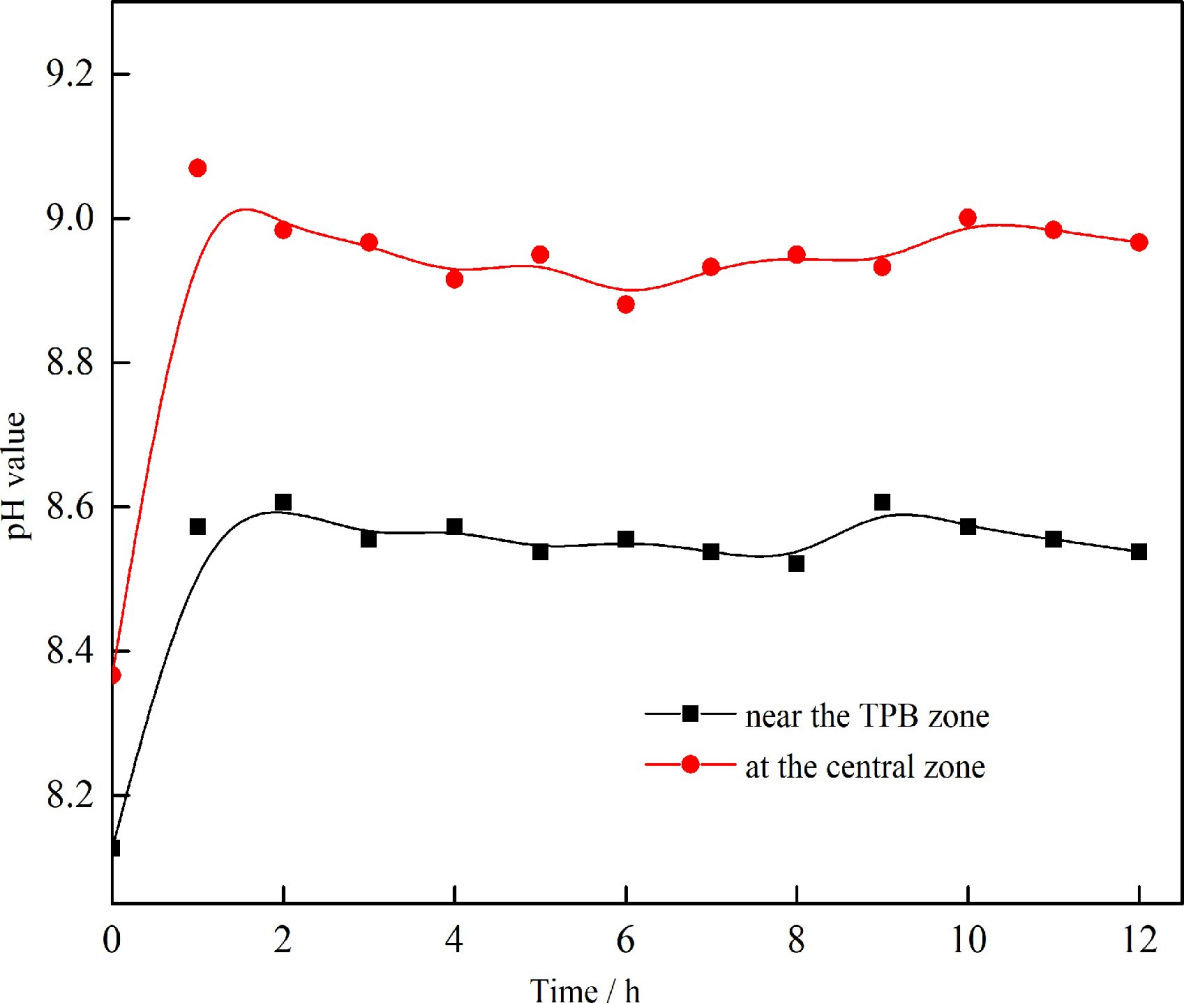
Variations of pH values with the exposure time.

To understand the corrosion behavior of the AZ91D alloy under the heterogeneous electrolyte layer, the pH values were measured at the center and near the TPB regions. As shown in Fig 6, both pH values increased in the first hour and remained almost stable during the remaining time. The cathodic reaction on Mg alloy in the atmosphere was thought to be due to water reduction and oxygen reduction (reactions 1 and 2), which resulted in the accumulation of hydroxyl ions and the increase of pH at the initial stage [4]. At the same time, carbon dioxide in the environment, which acts as an acidic gas, was expected to have a pronounced effect owing to a limited amount of electrolyte. When dissolved in water, carbon dioxide can form carbonic acid (reaction 3). In alkaline conditions, carbonic acid forms carbonate (reactions 4 and 5), leading to the consumption of hydroxyl ions [16]. Owing to the neutralization effect of carbon dioxide gas, the pH value remained stable for a long time. At the same time, it should be noted that the pH value was lower near the TPB zone than that at the center, which could be attributed to the rapid production of carbonate and fast consumption of hydroxyl ions for the short diffusion path of carbon dioxide near the TPB zone.

$${2H}_{2}O+2e^{-}\to 2OH^{-}+H_{2}$$ (1)

$${O_{2}+2H}_{2}O+4e^{-}\to 4OH^{-}$$ (2)

$${{CO}_{2}}_{\left(aq\right)}+H_{2}O\to H_{2}{{CO}_{3}}_{\left(aq\right)}$$ (3)

$$H_{2}{CO}_{3}+{OH}^{-}\to {HCO}_{3}^{-}+H_{2}O$$ (4)

$${HCO}_{3}^{-}+{OH}^{-}\to {CO}_{3}^{2-}+H_{2}O$$ (5)

### 3.3 Surface analysis

**Fig 7:**
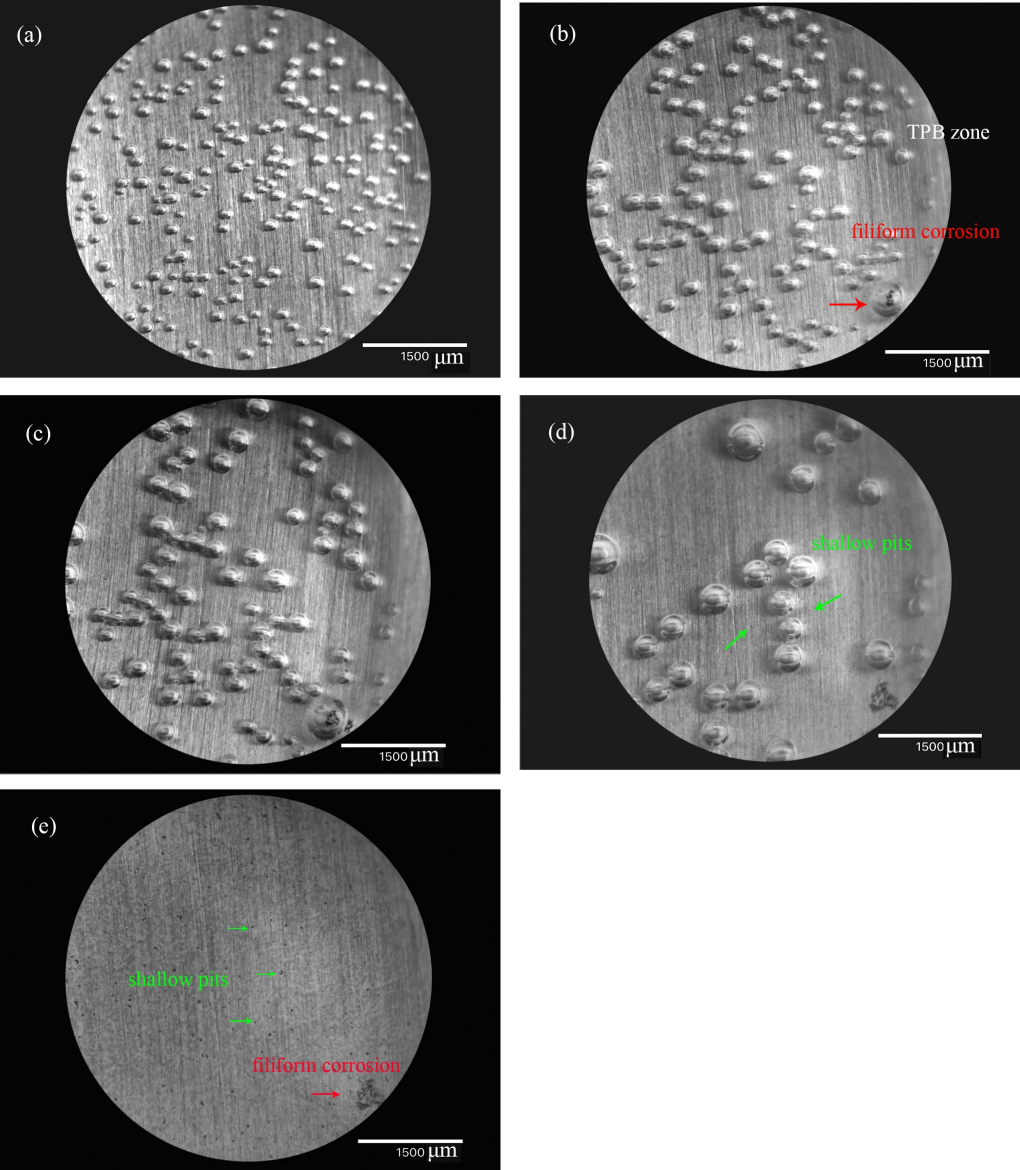
Optical images of AZ91D alloy under TEL after different time. a: 5min, b: 3 h, c: 6 h, d: 10 h.

Fig 7 shows optical images of the AZ91D alloy under TEL. Soon after the placement of the electrolyte layer, hydrogen bubbles were observed randomly adhering to the surface (Fig 7A). After several hours, a number of shallow pits appeared on the surface of AZ91D alloy, with the size ranged from several to dozens of microns. At the same time, serious corrosion was observed near the TPB zone, as shown in Fig 7B. With increasing time, the corrosion features propagated from the initiation pit and took the form of dark filament near the TPB zone (Fig 7C and 7D). The head of the filament expanded forward quickly, accompanied with releasing of voluminous hydrogen bubbles and leaving inert pits behind.

**Fig 8:**
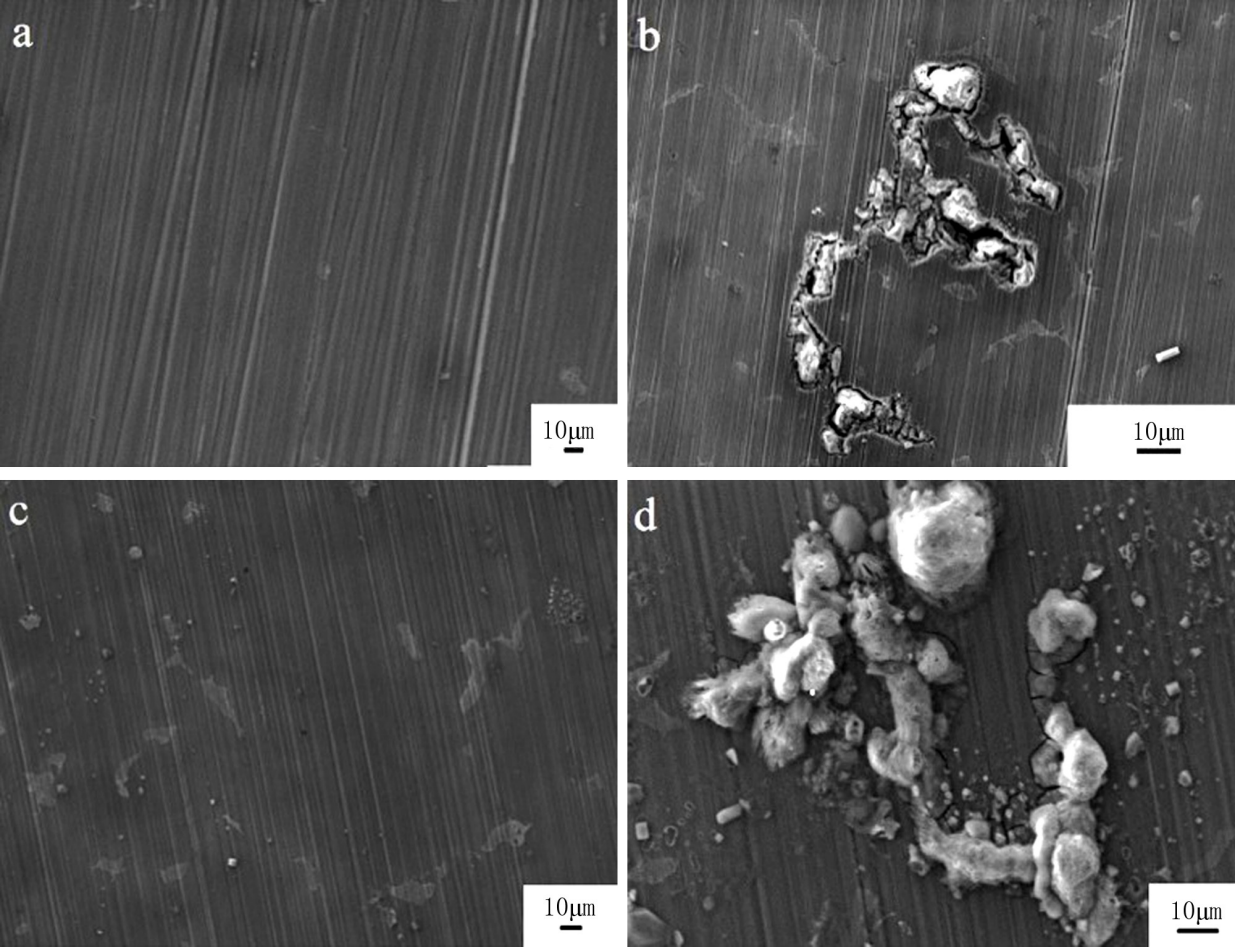
SEM images of AZ91D alloy after different exposure time. a: 1 h, at the central zone; b: 1 h, near the TPB zone; c: 6 h, at the central zone; d: 6 h, near the TPB zone.

The morphologies of different zones on the surface of the AZ91D alloy under the TEL are illustrated in Fig 8. After exposure for 1 hour, several scattered corrosion products appeared at the central zone (Fig 8A). Near the TPB zone, some discontinuous curved cracks were observed with insoluble particles deposited (Fig 8B). The particles were irregular and discontinuous, with a size of several microns. After exposure for 6 hours, a certain number of irregular corrosion products were found to be dispersed at the center (Fig 8C). In the meantime, long curved tracks propagated along the surface and were covered by cellular corrosion product crusts near the TPB zone, as shown in Fig 8D. This filiform-like corrosion preferred to initiate and propagate near the TPB zone, different from the corrosion features at the center.

Mg alloys are typically multiphase materials, consisting of a dispersion of intermetallic particles in the α-Mg matrix [4] [17]. With the aid of EDS, the curved cracks were assumed and detected to be related to an intermetallic phase with more aluminum and less magnesium. This filiform corrosion might be attributed to the dissolution of α-phase Mg located around the intermetallic phase. Because of the significant difference in the nobility of the α-Mg and intermetallic phase, it is proposed that the intermetallic particles are the principal sites of cathodic activity, and this is coupled to the anodic attack of the surrounding Mg grains [18] [19] [20] [21].

The composition of major elements in the center and near the TPB zone for different times is shown in Table 1. Compared with the EDS data in the center, a depletion of magnesium and an enrichment in oxygen were found near the TPB zone. This can be attributed to the rapid anodic dissolution of Mg and the quick generation of corrosion products. With the propagation of corrosion, the content of magnesium further declined, accompanied by an increase of oxygen and carbon after 6 hours. This indicated the accumulation of insoluble corrosion products on the specimen surface.

**Table 1:** EDS data of the AZ91D alloy surface for the major elements (atomic %).

Time	Zone	Mg	O	C	Al	Zn	Mn
1 h	TPB zone	64.42	18.71	11.79	3.53	0.92	0.07
1 h	Center	74.12	11.24	11.31	2.87	0.59	0.02
6 h	TPB zone	45.39	30.37	18.26	1.80	0.48	0.06
6 h	Center	62.51	14.02	19.01	2.77	0.64	0.04

**Fig 9:**
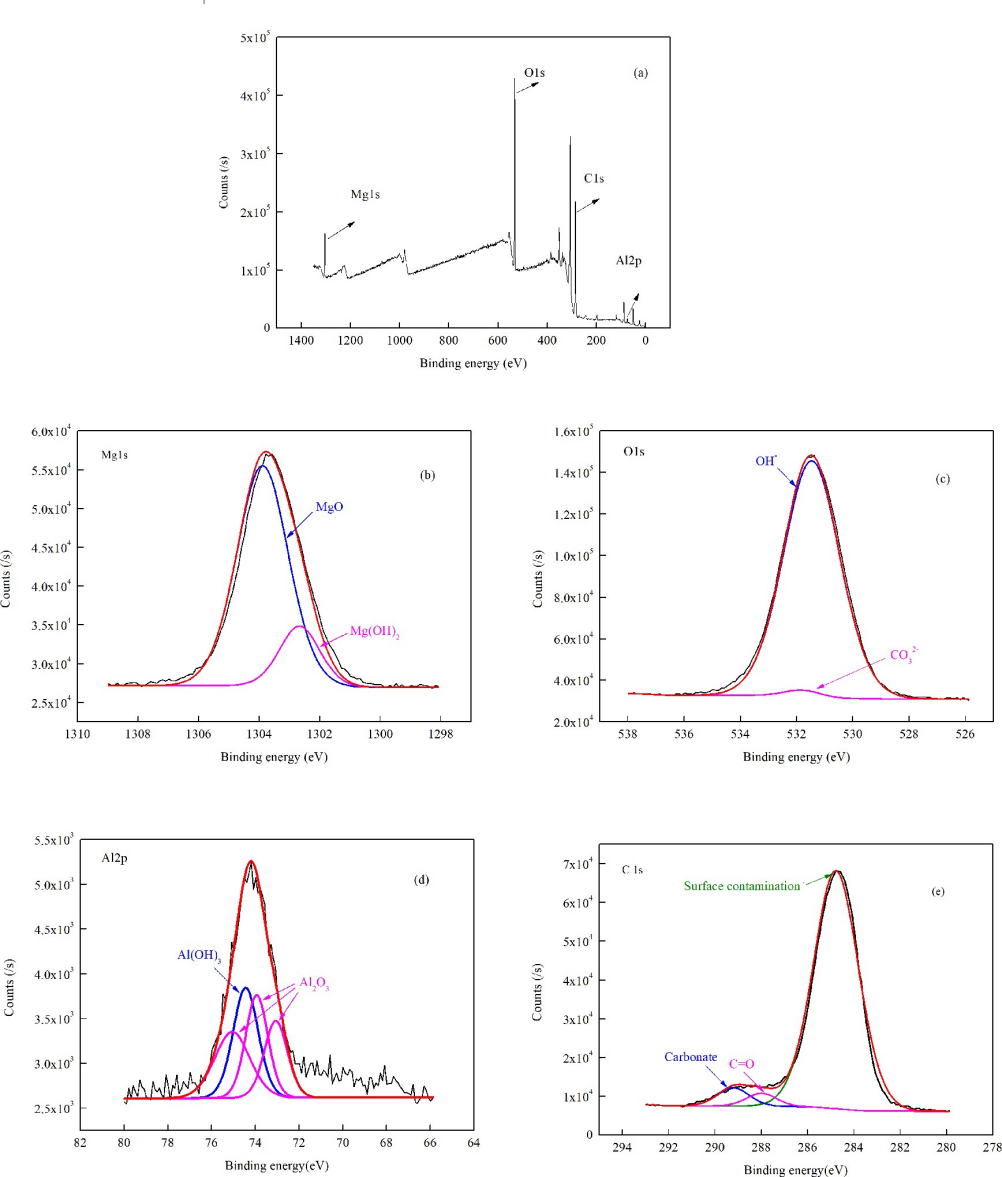
XPS analysis of corrosion products after exposure under TEL for 12 h. (a) general diagram, (b) Mg 1 s, (c) O 1s, (d) Al 2p, (e) C 1s.

After exposure under TEL for 12 h, the specimen was analyzed using XPS (Fig 9). As shown in Fig 9A, the XPS spectra of the specimen were obviously composed of Mg, O, C and Al elements peaks. The main corrosion products were MgO, Mg(OH)_2_, Al_2_O_3_ and Al(OH)_3_ (Fig 9B–9D). In the meantime, O1s spectrum showed the existence of OH^-^ and a small amount of CO_3_^2-^ (Fig 9C), indicating the conversion process of magnesium hydroxide to carbonate-containing corrosion products is relatively slow [22]. The analysis of the C1s spectrum (Fig 9E) showed that most of the carbon signal seemed to originate from atmospheric contamination or carbonate [23]. The C1s peak located at 284.8 eV corresponded to the carbon contamination on the surface. And the peak at 289.3 eV attributed to the presence of carbonate, which can verify the statement of carbonic acid involved in the corrosion process.

## 4 Discussion

Metals normally oxidize rapidly when exposed to atmosphere. After a short time, a very thin layer of MgO has formed. Under the electrolyte layer, some electrochemical corrosion cells formed on the surface of the AZ91D alloy, which resulted in the random production of magnesium ions at local anodes and hydroxyl ions at local cathodes. Accompanied by the migration of these ions, magnesium hydroxide corrosion product was generated and deposited on the surface. The corrosion products contained considerable amounts of insoluble MgO and Mg(OH)_2_.

**Fig 10:**
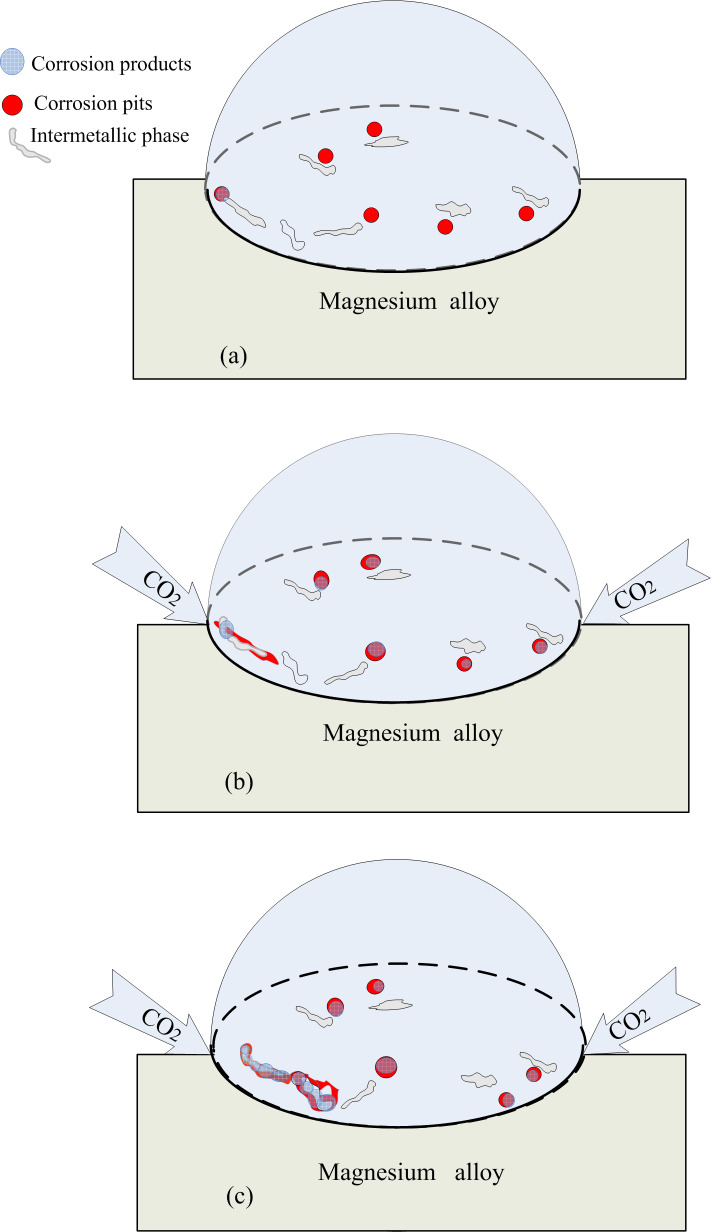
Schematic illustration of corrosion mechanism for Mg alloys under a heterogeneous electrolyte layer.

The schematic illustration of corrosion mechanism for Mg alloys under a heterogeneous electrolyte layer was shown in Fig 10. Comparatively, the supply and diffusion of carbon dioxide was easier near the TPB zone, leading to the fast consumption of hydroxyl ions and a low pH value. It could be deduced that the corrosion products were less stable near these zones, and the anodic dissolution rate of magnesium was high. At the same time, the migration and accumulation of chloride ions at the anodic sites enhanced the dissolution process of magnesium and caused serious corrosion to occur near the TPB zone (Fig 10A).

With the progress of corrosion, the accumulation of the less soluble corrosion products provided a partly protective layer on the surface and inhibited corrosion by physically blocking the anodic sites. Moreover, magnesium hydroxides and magnesium oxides are non-conducting and cannot serve as cathodes. Therefore, the propagation of active anodes cannot go deeply under the less soluble product layer but can only expand along the surface. Because the stability of the corrosion product layer was weak at low pH values, the active anodes preferred to propagate near the TPB zone and moved forward to consume the intact surface. After a period of time, filiform corrosion developed near the TPB zone under the electrolyte layer (Fig 10B and 10C).

It should be noted that the existence of TPB has important influences on the corrosion locations, the corrosion morphologies and the corrosion rate of the Mg alloy. The TPB zone showed an impact on the anodic dissolution process of the Mg alloy, which was different from that of carbon steel. For the corrosion of carbon steel, the TPB influences the cathodic reduction process by facilitating the mass transport of oxygen. However, here, TPB had an impact on the anodic reaction of Mg alloy by assisting the mass transport process of carbon dioxide, which lowered the pH value and accelerated the anodic dissolution process.

## 5 Conclusion

The corrosion behavior of an AZ91D magnesium alloy was investigated under a heterogeneous electrolyte layer, and the following conclusions were obtained.

1. Under a heterogeneous electrolyte layer, a number of shallow pits were formed randomly on the surface of magnesium alloy. At the same time, serious localized corrosion preferred to initiate and propagate near the TPB zone, which resulted in the formation of filiform corrosion on the surface of the Mg alloy.
2. The TPB zone played a vital role in determining the locations of localized corrosion, the corrosion morphologies and the corrosion rate. The TPB zone mainly influenced the anodic corrosion process of the magnesium alloy by facilitating the mass transport process of carbon dioxide, which lowered the pH value.
